# Exopolysaccharides enriched in rare sugars: bacterial sources, production, and applications

**DOI:** 10.3389/fmicb.2015.00288

**Published:** 2015-04-10

**Authors:** Christophe Roca, Vitor D. Alves, Filomena Freitas, Maria A. M. Reis

**Affiliations:** ^1^Research Unit on Applied Molecular Biosciences, Rede de Química e Tecnologia, Departamento de Química, Faculdade de Ciências e Tecnologia, Universidade Nova de Lisboa, Caparica, Portugal; ^2^Linking Landscape, Environment, Agriculture and Food, Instituto Superior de Agronomia, Universidade de Lisboa, Lisboa, Portugal

**Keywords:** bacterial extracellular polysaccharides, rare-sugars, fucose, rhamnose, glucuronic acid

## Abstract

Microbial extracellular polysaccharides (EPS), produced by a wide range of bacteria, are high molecular weight biopolymers, presenting an extreme diversity in terms of chemical structure and composition. They may be used in many applications, depending on their chemical and physical properties. A rather unexplored aspect is the presence of rare sugars in the composition of some EPS. Rare sugars, such as rhamnose or fucose, may provide EPS with additional biological properties compared to those composed of more common sugar monomers. This review gives a brief overview of these specific EPS and their producing bacteria. Cultivation conditions are summarized, demonstrating their impact on the EPS composition, together with downstream processing. Finally, their use in different areas, including cosmetics, food products, pharmaceuticals, and biomedical applications, are discussed.

## Introduction

Bacterial extracellular polysaccharides (EPS) presenting a wide range of physicochemical properties have emerged as promising polymers for many commercial applications in different industrial sectors like food, pharmaceuticals, cosmetics, oil drilling, and paper manufacturing (Kumar and Mody, 2009; Freitas et al., 2011a). Despite this potential, bacterial EPS currently represent a very small fraction of the global polymer market, mostly because of their production costs. However, in some cases, they might address very specific niche markets, where the production cost does not become a limiting step for their commercialization (Freitas et al., 2011a).

Rare sugars are monosaccharides that are not commonly found in nature, where D-glucose, D-galactose, D-fructose, D-xylose, D-ribose, and L-arabinose are more abundant. Rare sugars such as L-fucose, L-rhamnose, or uronic acids present many interesting properties, making them attractive for various fields of applications, such as anti-inflammatory substances, antioxidant, or as building blocks to synthesize the nucleoside analogs which are used as antiviral agents, and justifying the effort to produce them synthetically (Kumar et al., 2007). Today, some of them might be produced biochemically by the use of specialized enzymes belonging usually to the classes of keto–aldol isomerases, epimerases, and oxidoreductases using glucose as main precursor (Granström et al., 2004; Beerens et al., 2012). Their scarceness makes them highly valuable and, consequently, bacterial EPS containing rare sugars represent an interesting source for their isolation and production. Although polysaccharides containing rare sugars are also found in plants, seaweeds, and animals, microbial production of such polymers is advantageous for several reasons, namely, production is not affected by environmental factors, the bioprocesses are easily controlled and manipulated, and they guaranty fast and reproducible production.

**FIGURE 1:**
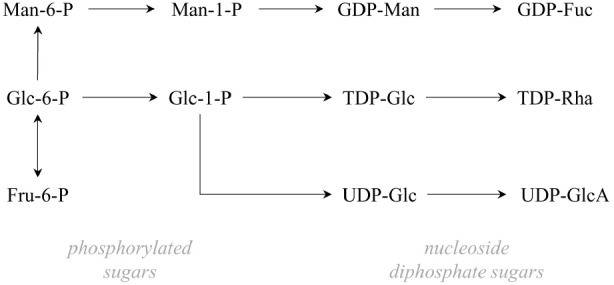
**Schematic diagram of the biosynthetic pathways in Gram-negative bacteria involved in the intracellular synthesis of nucleoside diphosphate sugars that are precursors of some rare sugar monomers (Fuc, fucose; Glc, glucose; GlcA, glucuronic acid; Man, mannose; Rha, rhamnose; Fru, fructose; GDP, guanosine diphosphate; TDP, tyrosine diphosphate; UDP, uridine diphosphate)**.

Some EPS containing rare sugars have been extensively studied (Table 1), not only because of their composition, but also because of their physical and bioactive properties. These include polymers containing fucose, such as colanic acid (Ratto et al., 2006), fucogel (Paul et al., 1996), clavan (Vanhooren and Vandamme, 2000), or FucoPol (Freitas et al., 2011b), or rhamnose, such as rhamsan (Xu et al., 2014), gellan (Zhang et al., 2015), or welan gum (Wang et al., 2014; Figure 1).

**TABLE 1:** **Production and composition details of some bacterial polysaccharides rich in rare sugars**.

**EPS**	**Producing strain**	**Composition**	**Substrates**	**Production (g/L)**	**Productivity (g/L·day)**	**Reference**
Gellan gum	*Sphingomonas paucimobilis*	Rhamnose, glucose, and glucuronic acid	Sucrose	19.9–22.6	6.6–9.0	Zhang et al. (2015)
			Glucose	12.4	7.4	Giavasis et al. (2003)
			Whey	1.6–3.1	0.6–1.2	Dlamini and Peiris (1997)
		Acetyl and glyceryl groups	Soluble starch	37.5–43.6	14.5–18.8	Bajaj et al. (2006)
			Molasses	13.8	n.a.	Banik et al. (2007)
Welan	*Alcaligenes* sp.	Rhamnose, glucose, and glucuronic acid	Glucose	25.0–26.3	8.3–8.8	Li et al. (2011)
			Corn starch	22.8	7.6	Li et al. (2010)
Clavan	*Clavibacter michiganensis*	Fucose, glucose, and galactose	Glucose	0.7	0.06	van den Bulk et al. (1991)
		Acetyl, pyruvyl, and succinyl groups				
FucoPol	*Enterobacter* A47	Fucose, glucose, galactose, and glucuronic acid	Glycerol	7.5–8.0	2.0–2.5	Torres et al. (2012)
			Glucose	13.4	3.4	Freitas et al. (2014)
			Xylose	5.4	1.4	Freitas et al. (2014)
		Acetyl, pyruvyl, and succinyl groups				
Hyaluronic acid	*Streptococcus* sp.	Glucuronic acid and acetylglucosamine	Glucose	0.4–6.9	0.8–1.5	Huang et al. (2008)

Extensive work has been performed to discover new strains able to produce such rare sugar-rich EPS and to improve their production and purification processes. Many of those polymers are being studied for new applications in pharmaceuticals cosmetics, food products, among others. Rare sugar-rich EPS can also be used as sources of rare sugar monosaccharides. Such EPS can be subjected to hydrolysis (either enzymatic or chemical) and the resulting monomers can be separated by chromatographic methods to yield pure monosaccharides. The rare sugars thus obtained can then be used as precursors for the synthesis of high-value molecules for use in high-value applications. The exploitation of EPS as sources of rare sugars still remains a rather unexplored and unexploited aspect. The economic viability of such strategy depends on the EPS content on a given rare sugar but the high demand for these molecules will be a strong driving force.

## EPS Containing Rare Sugars and Their Producers

Even though not all rare sugars can be found in microbial polysaccharides, some EPS, secreted by bacteria in specific limited conditions, contain rare sugars, offering an opportunity to produce them in sustainable and controlled conditions. These include polymers containing fucose, such as colanic acid, fucogel, and clavan, or rhamnose, such as rhamsan, gellan, diutan, or welan gum, very often in combination with uronic acids such as glucuronic or galacturonic acids (Kumar and Mody, 2009).

Similarly to most bacterial EPS, rare sugar enriched EPS synthesis occurs intracellularly (Freitas et al., 2011a,b). Energy-rich monosaccharides, mainly nucleoside diphosphate sugars (NDP-sugars), are synthesized from phosphorylated sugars formed from primary metabolites (Figure 1). Polymerization commonly occurs at the cytoplasmic face of the inner membrane or at the periplasm, and the polysaccharides are exported to the extracellular.

The presence of rare sugars on bacterial EPS is not well understood yet, but they may provide the cells with an extra biological protection in addition to the physical barrier provided by the EPS.

### Rhamnose Containing EPS

Most rhamnose containing EPS belong to the family of sphingans are secreted by strains of the *Sphingomonas* genus (Fialho et al., 2008). Sphingans, as rhamsan, gellan, diutan, or welan gum present a common linear tetrasaccharide repeating unit Glc-GlcA-Glc-Rha or Man (Pollock, 1993). The presence of rhamnose or mannose at the end the repeating unit is responsible for the main structural variations between sphingans. For instance, diutan has exactly the same backbone as gellan (Glu-GlcA-Glu-Rha) but is substituted with Rha-Rha side chain. *Klebsiella* genus also presents EPS producers. For instance, *Klebsiella* I-714 produces an EPS with a high rhamnose content, composed of hexasaccharide units of L-rhamnose, D-galactose, and D-glucuronic acid in a molar ratio of 3:2:1 (Serrat et al., 1995).

### Fucose Containing EPS

Clavan, composed of tetrasaccharide repeating units of D-glucose, D-galactose, L-fucose, and pyruvic acid in the molar ratio of 1:1:2:1, is one of the richest polymers in fucose. It is produced by *Clavibacter* strains, in particular *C. michiganensis* (Vanhooren and Vandamme, 2000). In comparison, Fucogel, commercialized by Solabia (France) is a linear anionic polymer produced by *K. pneumoniae*, constituted of trisaccharide repeating units of galacturonic acid, L-fucose, and D-galactose (Vanhooren and Vandamme, 2000). A marine bacterium *Enterobacter cloacae* was also reported to produce a polymer composed fucose, galactose, glucose, and glucuronic acid in a molar ratio of 2:1:1:1 (Iyer et al., 2005). The strain *Enterobacter* A47 synthesizes FucoPol, a fucose-containing polysaccharide with nearly equimolar amounts of fucose, glucose, and galactose, and minor content of glucuronic acid (10–15 mol%; Freitas et al., 2011b).

### Other Bacterial EPS

Even rarer sugars that rhamnose and fucose might be encountered in bacterial EPS. For instance, strains of the rumen bacterium *Butyrivibrio fibrisolvens* produce significant amounts of EPS containing L-altrose and L-iduronic acid (Ferreira et al., 1997). Due to its scarcity, reports on altrose production and applications remain still limited today. *Pseudomonas viscogena* produces a polysaccharide containing another scarce sugar, allose, the C2 epimer of altrose, but only in small proportion (around 2.5% of sugar content; Takemoto and Igarashi, 1984). A much more studied rare sugar containing EPS is hyaluronic acid (HA), because of its proven properties (Collins and Birkinshaw, 2013). HA is a linear polymer composed of *N*-acetylglucosamine and glucuronic acid, as disaccharidic repeating units produced by *Streptococcus zooepidemicus* (Liu et al., 2011)

## Bioproduction

### Cultivation Conditions

Bacterial EPS are industrially produced in single strain systems (Kumar and Mody, 2009). As for other bacterial EPS production bioprocesses, the production of EPS rich in rare sugars rely on the use of carbohydrates as carbon sources because they allow for high productivities and yields (Table 1; Kumar et al., 2007). Sucrose and glucose are the most commonly used substrates, while xylose, galactose and lactose are less frequently used because many bacteria are unable to use them or they result in reduced polymer productivities (Singh et al., 2008; Zhang and Chen, 2010). Since the substrate cost accounts for up to 40% of the total production costs of microbial polymers (Kumar and Mody, 2009), there is an intensive search for alternative raw materials to reduce the overall production costs. For that reason, several inexpensive agricultural and industrial wastes and by-products have been proposed as substrates for microbial cultivation, including lignocellulosic materials, cheese whey, molasses, and glycerol-rich product (Table 1). On the other hand, the use of some agricultural and industrial wastes/by-products is held up by the difficulty in guarantying their supply in terms of both quantity and quality, and they may require costly pretreatments prior to use. Moreover, non-reacted components may accumulate in the broth and eventually be carried-over to the final product. Hence, for applications wherein high-purity and high quality products are needed, the use of wastes or by-products may not be an option or, otherwise, higher investment must be put in downstream procedures (Freitas et al., 2011a).

Media composition and cultivation conditions highly influence the amount of polysaccharide synthesized, as well as the final product’s composition, molecular structure, average molecular weight, and, consequently, their functional properties (Nicolaus et al., 2010; Poli et al., 2011). Hence, it is possible to manipulate polysaccharides’ characteristics by altering the growth conditions of the producing strains, thus obtaining biopolymers with tailored properties to fit a given application. Enrichment of the polysaccharides in rare sugar monomers is of particular interest since it may enhance their bioactive properties, thus increasing their market value. However, the impact of media and cultivation conditions on EPS composition is strain-dependent and only a few bacteria can be manipulated to alter the composition of the EPS they synthesize. In fact, for most bacteria, EPS sugar composition is a genetically determined trait, which is not significantly altered by the cultivation conditions (López et al., 2003; Celik et al., 2008). An exception to this is, for example, the bacterium *Enterobacter* A47 that was shown to be able to synthesize EPS with different fucose contents by cultivation as a function of the carbon source used and the operating conditions (Torres et al., 2012; Freitas et al., 2014). High fucose content EPS (36–38 mol%) were synthesized by *Enterobacter* A47 using glycerol or xylose as sole carbon sources, while glucose, methanol or citrate led to lower contents (22–29 mol%; Freitas et al., 2014). On the other hand, the composition of the EPS produced by *Enterobacter* A47 could also be manipulated by cultivation at different pH and/or temperature ranges: maximum fucose content (>30 mol%) was obtained for cultivation at 25–35°C and pH = 6.0–8.0 (Torres et al., 2012).

### Downstream Processing

The recovery of bacterial EPS from the culture broth commonly involves cell removal, usually by centrifugation or filtration, polymer precipitation from the cell-free supernatant by the addition of a precipitating agent consisting of a water-miscible solvent in which the polymer is insoluble (e.g., methanol, ethanol, isopropanol, acetone) and drying of the precipitated polymer (e.g., by freeze drying, drum drying; Freitas et al., 2011a, 2013). For some applications that require high purity grade products, the polymer is subjected to one or several additional methods, such as re-precipitation of the polymer from diluted aqueous solution, deproteinization by chemical or enzymatic methods, and membrane processes, such as dialysis, ultrafiltration and diafiltration (Kumar et al., 2007; Wang et al., 2007; Ayala-Hernández et al., 2008; Bahl et al., 2010; Freitas et al., 2011a). The most appropriate downstream procedure is selected to guaranty the required products’ purity. Additionally, the impact of the purification procedures on the polysaccharides’ properties must also be taken in consideration.

## Industrial and Biomedical Applications

Most polysaccharides’ applications are related to their behavior in aqueous media. Their physical and chemical characteristics, such as water binding capacity, high average molecular weight, polyelectrolyte behavior (in some cases), molecular structure, and the possibility of being chemically modified, enables this type of molecules to present diverse functional properties (e.g., thickening, film forming, gelling, emulsion stabilizing, flocculating, and nano/microstructures production abilities; Dumitriu, 2004). Furthermore, in the case fucose and rhamnose-rich bacterial polysaccharides, they also present interesting biological activities that render these molecules potential to be used in a wide range of applications, particularly in added value products like cosmetics, pharmaceuticals, medical devices, and functional food products (Péterszegi et al., 2003a; Ravelojaona et al., 2009). As examples, rhamnose is commonly used as precursor for the production of aroma and flavors and, together with fucose, has attracted more attraction as fucose and rhamnose-rich oligo- and polysaccharides (FROP and RROP, respectively) have been found to counteract several of the mechanisms involved in skin aging (Robert et al., 2009).

Gellan gum is a linear anionic EPS, and its native form contains two acyl substituents, L-glyceryl and acetyl, being known as high acyl gellan (HA-gellan). The substituents may be removed by alkaline hydrolysis to give deacetylated gellan, also called low acyl gellan (LA-gellan). HA-gellan usually produces elastic, soft, non-brittle, and opaque gels while LA-gellan enables the formation of non-elastic, hard, brittle, and transparent gels (Sworm, 2009). Therefore, a wide range of structures, with varied rheological properties and appealing textures may be designed by controlling the acyl content. Gellan produced by cultivation of a pure culture of *Sphingomonas elodea* using a sugar as carbon source, and commercialized by CPKelco (with the trade name KELCOGEL^®^), has been used in the food industry as additive that functions, not only as gelling, but also as texturizing, stabilizing, suspending, film-forming, and structuring agent. Types of food products that contain gellan gum include bakery fillings, dairy products, low-fat spreads, dessert gels, jams and jellies, sauces, and structured foods (Freitas et al., 2013; Prajapati et al., 2013). It has also been used to develop edible coatings for the improvement of fruits shelf life (Rojas-Graü et al., 2007; Tapia et al., 2008).

The applications of gellan gum in pharmaceutical technology and medicine were recently reviewed by Osmałek et al. (2014). In pharmaceuticals, it has been studied as carrier material in drug delivery, in the form of tablets, capsules, beads, and hydrogels. Formulations based on gellan gum for oral, ophthalmic, and nasal applications have been developed. In medicine field, the use of gellan has been investigated for tissue engineering (e.g., for cartilage reconstruction and guided bone regeneration) and wound healing (e.g., in wound dressings to inhibit postsurgical adhesion and scar formation). Gellan sulfate derivatives are promising materials for rheumatoid arthritis treatment, as they have a tendency for selective binding of fibronectin molecules (Miyamoto et al., 2001), and for the development of cell-hybrid materials for artificial veins design due to the anticoagulant activity of such derivatives (Miyamoto et al., 2002).

Regarding welan gum, the main applications studied so far are in the field of cement production. The use of low viscosity welan gum in cement compositions reduces fluid loss of those compositions, increases the suspension properties of cement suspensions, being effective at low concentrations (Kaur et al., 2014). The rheological properties of welan gum in aqueous media were compared to that of xanthan gum, well known for its good thickening capacity. For the same biopolymer concentration, welan solutions presented higher apparent viscosity and higher viscoelastic moduli (loss and storage moduli). In addition, the welan gum solution is able to maintain high viscosity at high temperature while the molecular aggregation of xanthan gum is more sensitive to temperature variations (Xu et al., 2013). As such, it is envisaged the application of welan gum in the same areas as xanthan gum, such as in food products (e.g., jellies, beverages, dairy products, and salad dressings); as well as in oil drilling fluids. As welan gum is a rhamnose containing polysaccharide, attention should be driven to the study of its biological activity and applications in the cosmetic, pharmaceutical, and medicine fields.

Native rhamsan gum is non-gel forming polysaccharide, but originates thermostable highly viscous solutions even at temperatures above 100°C. It possesses good stability under shearing, great compatibility with high salt concentrations and excellent suspension properties, even superior to that of xanthan gum. Furthermore, gelation was observed in deacetylated rhamsan gum solution at concentrations of 0.3%wt at low temperature (Tako et al., 2003). This polymer finds applications in the same fields of the other polysaccharides of the gellan family, namely in the oil field, concrete, food products, cosmetics, pharmaceuticals, and medicine (e.g., plastic surgery), referred by Xu et al. (2014).

Fucose is another rare sugar that is today used in the composition of anticarcinogenic and anti-inflammatory drugs, in the preparation of creams for the acceleration of wound healing and as hydrating and anti-aging additives (Péterszegi et al., 2003a,b; Cescutti et al., 2005). Fucogel, a fucose containing EPS, has been used extensively in skin care cosmetic formulations (Bauer et al., 2012; Tecco and Sanders, 2012). This fact is mainly due to its moisturizing properties and to the scientific evidence of the action of fucogel and fucogel oligosaccharides as skin anti-aging agents, namely for stimulation of fibroblast proliferation and survival (Péterszegi et al., 2003a). Another recently reported fucose-rich EPS, FucoPol, has demonstrated interesting functional characteristics, such as thickening, emulsion stabilizing, film forming, and flocculating capacities (Cruz et al., 2011; Freitas et al., 2011b; Ferreira et al., 2014). These properties envisage its application in several areas, namely in emulsion-based cosmetics, drug delivery systems, biodegradable films for packaging, oil drilling fluids, and as biodegradable bioflocculant (e.g., in wastewater treatment).

Hyaluronic acid is abundantly found in mammalian skin where it constitutes a high fraction of the extracellular matrix of the dermis. Its physical and biochemical properties, either in solution or as hydrogel, are attractive for various technologies related to body repair. As such, it is a biomaterial of increasing importance finding applications in cosmetics, pharmaceuticals, and medicine (Collins and Birkinshaw, 2013). Either in a stabilized form or in combination with other polymers, it is used in cosmetic surgery as a component of commercial dermal fillers (e.g., Dermalive^®^ and Hylaform^®^). It is reported that injection of such products into the dermis, can reduce facial lines and wrinkles in the long term (Brown and Jones, 2005). HA-based materials have been applied in scaffolds for wound healing, bone and cartilage tissue repair and regeneration, nerve and brain tissue repair, soft tissue repair, and smooth muscle engineering (Collins and Birkinshaw, 2013). In addition, this biopolymer has been extensively explored as drug carrier, including target specific and long-acting delivery of protein, peptide, and nucleotide therapeutics (Oh et al., 2010).

## Conclusion

Only a minority of microbial EPS are today used as commodity products mostly because of their production and purification costs. However, the presence of rare sugars in specific EPS may confer them high added value, to be used in highly specialized applications such as biomedicine, pharmaceuticals, or cosmetics. The physical and chemical properties of the polymers determine their potential applications as thickeners, film producers, emulsion stabilizers, flocculants or materials for nano/microparticles, and scaffolds for tissue engineering among others. In addition to that, their biological activity, potentiated by the occurrence of rare sugars, may offer new market opportunities. EPS microbial production presents the advantage of being simple, robust, reproducible, and easily controllable by growth conditions, such as type and concentration of carbon source, pH, or temperature. However, despite the fact it is possible to produce a polymer with constant characteristics (e.g., composition, purity, and homogeneity), a better understanding of EPS synthesis, in particular EPS with high rare sugars content, is still needed to maximize production and obtain an economical viable process.

### Conflict of Interest Statement

The authors declare that the research was conducted in the absence of any commercial or financial relationships that could be construed as a potential conflict of interest.

## References

[B1] Ayala-Hernández I. Hassan A. Goff H. D. Mira de Orduña R. Corredig M. (2008). Production, isolation and characterization of exopolysaccharides produced by *Lactococcus lactis* subsp. *cremoris* JFR1 and their interaction with milk proteins: effect of pH and media composition. Int. Dairy J. 18, 1109–1118 10.1016/j.idairyj.2008.06.008

[B2] Bahl M. A. Schultheis E. Hempel D. C. Nörtemann B. Franco-Lara E. (2010). Recovery and purification of the exopolysaccharide PS-EDIV from *Sphingomonas pituitosa* DSM 13101. Carbohydr. Polym. 80, 1037–1041 10.1016/j.carbpol.2010.01.021

[B3] Bajaj I. B. Saudagar P. S. Singhal R. S. Pandey A. (2006). Statistical approach to optimization of fermentative production of gellan gum from *Sphingomonas paucimobilis* ATCC 31461. J. Biosci. Bioeng. 102, 150–156. 10.1263/jbb.102.150 17046526

[B4] Banik R. M. Santhiagu A. Upadhyay S. N. (2007). Optimization of nutrients for gellan gum production by *Sphingomonas paucimobilis* ATCC 31461 in molasses based medium using response surface methodology. Bioresour. Technol. 98, 792–797. 10.1016/j.biortech.2006.03.012 16707262

[B5] Bauer A. Dörschner A. Filbry A. Göppel A. Lanzendörfer G. Schneider K. (2012). Cosmetic or Dermatological Stick. U.S. Patent No 8,329,200 B2. Washington, DC: U.S. Patent and Trademark Office.

[B6] Beerens K. Desmet T. Soetaert W. (2012). Enzymes for the biocatalytic production of rare sugars. Ind. Microbiol. Biotechnol. 39, 823–834. 10.1007/s10295-012-1089-x 22350065

[B7] Brown M. Jones S. (2005). Hyaluronic acid: a unique topical vehicle for the localized delivery of drugs to the skin. J. Eur. Acad. Dermatol. 19, 308–318. 10.1111/j.1468-3083.2004.01180.x 15857456

[B8] Celik G. Y. Aslim B. Beyatli Y. (2008). Characterization and production of the exopolysaccharide (EPS) from *Pseudomonas aeruginosa* G1 and *Pseudomonas putida* G12 strains. Carbohydr. Polym. 73, 178–182 10.1016/j.carbpol.2007.11.021

[B9] Cescutti P. Kallioinen A. Impallomeni G. Toffanin R. Pollesello P. Leisola M. (2005). Structure of the exopolysaccharide produced by *Enterobacter amnigenus*. Carbohydr. Res. 340, 439–447. 10.1016/j.carres.2004.12.008 15680599

[B10] Collins M. N. Birkinshaw C. (2013). Hyaluronic acid based scaffolds for tissue engineering—a review. Carbohydr. Polym. 92, 1262–1279. 10.1016/j.carbpol.2012.10.028 23399155

[B11] Cruz M. Freitas F. Torres C. A. V. Reis M. A. M. Alves V. D. (2011). Influence of temperature on the rheological behavior of a new fucose-containing bacterial exopolysaccharide. Int. J. Biol. Macromol. 48, 695–699. 10.1016/j.ijbiomac.2011.02.012 21376748

[B12] Dlamini A. M. Peiris P. S. (1997). Production of exopolysaccharide by *Pseudomonas* sp. ATCC 31461 (*Pseudomonas elodea*) using whey as fermentation substrate. Appl. Microbiol. Biotechnol. 47, 52–57 10.1007/s002530050887

[B13] Dumitriu S. (2004). Polysaccharides: Structural Diversity and Functional Versatility. Boca Raton: CRC Press 10.1201/9781420030822

[B14] Ferreira A. R. Torres C. A. V. Freitas F. Reis M. A. M. Alves V. D. Coelhoso I. M. (2014). Biodegradable films produced from the bacterial polysaccharide FucoPol. Int. J. Biol. Macromol. 71, 111–116. 10.1016/j.ijbiomac.2014.04.022 24769364

[B15] Ferreira F. Kenne L. Cotta M. A. Stack R. J. (1997). Structural studies of the extracellular polysaccharide from *Butyrivibrio fibrisolvens* strain CF3. Carbohydr. Res. 30, 193–203 10.1016/S0008-6215(97)00097-9 9232840

[B16] Fialho A. M. Moreira L. M. Granja A. T. Popescu A. O. Hoffmann K. Sá-Correia I. (2008). Occurrence, production, and applications of gellan: current state and perspectives. Appl. Microbiol. Biotechnol. 79, 889–900. 10.1007/s00253-008-1496-0 18506441

[B17] Freitas F. Alves V. D. Coelhoso I. Reis M. A. M. (2013). “Production and food applications of microbial biopolymers,” in Engineering Aspects of Food Biotechnology. Part I: Use of Biotechnology in the Development of Food Processes and Products, eds Teixeira J. A. Vicente A. A. (Boca Raton: CRC Press/Taylor & Francis Group), 61–88.

[B18] Freitas F. Alves V. D. Gouveia A. R. Pinheiro C. Torres C. A. V. Grandfils C. (2014). Controlled production of exopolysaccharides from *Enterobacter* A47 as a function of carbon source with demonstration of their film and emulsifying abilities. Appl. Biochem. Biotechnol. 172, 641–657. 10.1007/s12010-013-0560-0 24104690

[B19] Freitas F. Alves V. D. Reis M. A. M. (2011a). Advances in bacterial exopolysaccharides: from production to biotechnological applications. Trends Biotechnol. 29, 388–398. 10.1016/j.tibtech.2011.03.008 21561675

[B20] Freitas F. Alves V. D. Torres C. A. V. Cruz M. Sousa I. Melo M. J. (2011b). Fucose-containing exopolysaccharide produced by the newly isolated *Enterobacter* strain A47 DSM 23139. Carbohydr. Polym. 83, 159–165 10.1016/j.carbpol.2010.07.034

[B21] Giavasis I. Robertson I. McNeil B. Harvey L. M. (2003). Simultaneous and rapid monitoring of biomass and biopolymer production by *Sphingomonas paucimobilis* using Fourier transform-near infrared spectroscopy. Biotechnol. Lett. 25, 975–979. 10.1023/A:1024040420799 12889834

[B22] Granström T. B. Takata G. Tokuda M. Izumori K. (2004). Izumoring: a novel and complete strategy for bioproduction of rare sugars. J. Biosci. Bioeng. 97, 89–94 10.1016/S1389-1723(04)70173-5 16233597

[B23] Huang W.-C. Chen S.-J. Chena T.-L. (2008). Production of hyaluronic acid by repeated batch fermentation. Biochem. Eng. J. 40, 460–464 10.1016/j.bej.2008.01.021

[B24] Iyer A. Mody K. Jha B. (2005). Characterization of an exopolysaccharide produced by a marine *Enterobacter cloacae*. Indian J. Exp. Biol. 43, 467–471. 15900914

[B25] Kaur V. Bera M. B. Panesar P. S. Kumar H. Kennedy J. F. (2014). Welan gum: microbial production, characterization, and applications. Int. J. Biol. Macromol. 65, 454–461. 10.1016/j.ijbiomac.2014.01.061 24508918

[B26] Kumar A. S. Mody K. (2009). “Microbial exopolysaccharides: variety and potential applications,” in Microbial Production of Biopolymers and Polymer Precursors: Applications and Perspectives, ed. Rehm B. H. M. (Norfolk: Caister Academic Press), 229–254.

[B27] Kumar A. S. Mody K. Jha B. (2007). Bacterial exopolysaccharides—a perception. J. Basic Microbiol. 47, 103–117. 10.1002/jobm.200610203 17440912

[B28] Li H. Xu H. Xu H. Li S. Ying H.-J. Ouyang P.-K. (2011). Enhanced welan gum production using a two-stage agitation speed control strategy in *Alcaligenes* sp. CGMCC2428. Bioprocess Biosyst. Eng. 34, 95–102. 10.1007/s00449-010-0450-6 20640447

[B29] Li S. Xu H. Li H. Guo C. (2010). Optimizing the production of welan gum by *Alcaligenes faecalis* NX-3 using statistical experiment design. Afr. J. Biotechnol. 9, 1024–1030.

[B30] Liu L. Liu Y. Li J. Du G. Chen J. (2011). Microbial production of hyaluronic acid: current state, challenges, and perspectives. Microb. Cell Fact. 10:99. 10.1186/1475-2859-10-99 22088095 PMC3239841

[B31] López E. Ramos I. Sanromán M. A. (2003). Extracellular polysaccharides production by *Arthrobacter viscosus*. J. Food Eng. 60, 463–467 10.1016/S0260-8774(03)00078-5

[B32] Miyamoto K. Asakawa Y. Arai Y. Shimizu T. Tokita M. Komai T. (2001). Preparation of gellan sulfate as an artificial ligand for removal of extra domain A containing fibronectin. Int. J. Biol. Macromol. 28, 381–385 10.1016/S0141-8130(01)00135-0 11325425

[B33] Miyamoto K. Kanemoto A. Hashimoto K. Tokita M. Komai T. (2002). Immobilized gellan sulfate surface for cell adhesion and multiplication: development of cell-hybrid biomaterials using self-produced fibronectin. Int. J. Biol. Macromol. 30, 75–80 10.1016/S0141-8130(02)00013-2 11911896

[B34] Nicolaus B. Kambourova M. Oner E. T. (2010). Exopolysaccharides from extremophiles: from fundamentals to biotechnology. Environ. Technol. 31, 1145–1158. 10.1080/09593330903552094 20718297

[B35] Oh E. J. Park K. Kim K. S. Kim J. Yang J. Kong J. (2010). Target specific and long-acting delivery of protein, peptide, and nucleotide therapeutics using hyaluronic acid derivatives. J. Control. Release 141, 2–12. 10.1016/j.jconrel.2009.09.010 19758573

[B36] Osmałek T. Froelich A. Tasarek S. (2014). Application of gellan gum in pharmacy and medicine. Int. J. Pharm. 466, 328–340. 10.1016/j.ijpharm.2014.03.038 24657577

[B37] Paul F. M. B. Perry D. F. Monsan P. F. (1996). Strain of Klebsiella pneumoniae, subsp. pneumoniae, and a Process for the Production of a Polysaccharide Containing L-Fucose. International Patent No. WO 9,623,057.

[B38] Péterszegi G. Fodil-Bourahla I. Robert A. M. Robert L. (2003a). Pharmacological properties of fucose. Applications in age-related modifications of connective tissues. Biomed. Pharmacother. 57, 240–245 10.1016/S0753-3322(03)00028-3 12888260

[B39] Péterszegi G. Isnard N. Robert A. M. Robert L. (2003b). Studies on skin aging. Preparation and properties of fucose-rich oligo- and polysaccharides. Effect on fibroblast proliferation and survival. Biomed. Pharmacother. 57, 187–194 10.1016/S0753-3322(03)00031-3 12888253

[B40] Poli A. Di Donato P. Abbamondi G. R. Nicolaus B. (2011). Synthesis, production, and biotechnological applications of exopolysaccharides and polyhydroxyalkanoates by Archaea. Archaea 2011:693253. 10.1155/2011/693253 22007151 PMC3191746

[B41] Pollock T. (1993). Gellan-related polysaccharides and the genus *Sphingomonas*. J. Gen. Microbiol. 139, 1939–1945 10.1099/00221287-139-8-1939

[B42] Prajapati V. D. Jani G. K. Zala B. S. Khutliwala T. A. (2013). An insight into the emerging exopolysaccharide gellan gum as a novel polymer. Carbohydr. Polym. 93, 670–678. 10.1016/j.carbpol.2013.01.030 23499110

[B43] Ratto M. Verhoef R. Suihko M.-L. Blanco A. Schols H. A. Voragen A. G. J. (2006). Colanic acid is an exopolysaccharide common to many enterobacteria isolated from paper-machine slimes. J. Ind. Microbiol. Biotechnol. 33, 359–367. 10.1007/s10295-005-0064-1 16418870

[B44] Ravelojaona V. Robert A. M. Robert L. (2009). Expression of senescence-associated β-galactosidase (SA-β-Gal) by human skin fibroblasts, effect of advanced glycation end-products and fucose or rhamnose-rich polysaccharides. Arch. Gerontol. Geriatr. 48, 151–154. 10.1016/j.archger.2007.12.004 18207583

[B45] Robert L. Labat-Robert J. Robert A.-M. (2009). Physiology of skin aging. Pathol. Biol. 57, 336–341. 10.1016/j.patbio.2008.09.007 19046830

[B46] Rojas-Graü M. A. Tapia M. S. Rodríguez F. J. Carmona A. J. Martin-Belloso O. (2007). Alginate and gellan-based edible coatings as carriers of antibrowning agents applied on fresh-cut Fuji apples. Food Hydrocoll. 21, 118–127 10.1016/j.foodhyd.2006.03.001

[B47] Serrat J. Caminal C. Godia F. Sola C. López-Santin J. (1995). Production and purification of rhamnose from microbial polysaccharide produced by *Klebsiella* sp. I-714. Bioprocess Eng. 12, 287–291 10.1007/BF00369505

[B48] Singh R. S. Saini G. K. Kennedy J. F. (2008). Pullulan: microbial sources, production and applications. Carbohydr. Polym. 73, 515–531 10.1016/j.carbpol.2008.01.003 26048217

[B49] Sworm G. (2009). “Gellan gum,” in Handbook of Hydrocolloids, eds O Philips G. Williams P. A. (Boca Raton, FL: CRC Press), 204–226 10.1533/9781845695873.204

[B50] Takemoto H. Igarashi T. (1984). Production of an Allose-containing Polysaccharide. US Patent 4.425.431.

[B51] Tako M. Tohma S. Taira T. Ishihara M. (2003). Gelation mechanism of deacetylated rhamsan gum. Carbohydr. Polym. 54, 279–285 10.1016/S0144-8617(03)00029-8

[B52] Tapia M. S. Rojas-Graü M. A. Carmona A. Rodríguez F. J. Soliva-Fortuny R. Martin-Belloso O. (2008). Use of alginate- and gellan-based coatings for improving barrier, texture and nutritional properties of fresh-cut papaya. Food Hydrocoll. 22, 1493–1503 10.1016/j.foodhyd.2007.10.004

[B53] Tecco M. A. Sanders C. (2012). Personal Skin Care Compositions Containing Anti-flammatory and Anti-microbial Agents. U.S. Patent No. 8,173,143 B2. Washington, DC: U.S. Patent and Trademark Office.

[B54] Torres C. A. V. Antunes S. Ricardo A. R. Grandfils C. Alves V. D. Freitas F. (2012). Study of the interactive effect of temperature and pH on exopolysaccharide production by *Enterobacter* A47 using multivariate statistical analysis. Bioresour. Technol. 119, 148–156. 10.1016/j.biortech.2012.05.106 22728195

[B55] van den Bulk R. W. Zevenhuizen L. P. T. M. Cordewener J. H. G. Dons J. J. M. (1991). Characterization of the extracellular polysaccharide produced by *Clavibacter michiganensis* subsp. michiganensis. Phytophatol 81, 619–623 10.1094/Phyto-81-619

[B56] Vanhooren P. T. Vandamme E. J. (2000). “Microbial production of clavan, an L-fucose rich polysaccharide,” in Food Biotechnology, eds Bielecki S. Tramper J. Polak J. (Amsterdam: Elsevier Science), 109–114.

[B57] Wang J. Ai H. Liu M. (2014). Enhanced welan gum production using cane molasses as substrate by *Alcaligenes* sp. ATCC31555. New Biotechnol. 31S. 10.1016/j.nbt.2014.05.1704

[B58] Wang X. Yuan Y. Wang K. Zhang D. Yang Z. Xu P. (2007). Deproteinization of gellan gum produced by *Sphingomonas paucimobilis* ATCC 31461. J. Biotechnol. 128, 403–407. 10.1016/j.jbiotec.2006.09.019 17069918

[B59] Xu L. Xu G. Liu T. Chen Y. Gong H. (2013). The comparison of rheological properties of aqueous welan gum and xanthan gum solutions. Carbohydr. Polym. 92, 516–522. 10.1016/j.carbpol.2012.09.082 23218329

[B60] Xu X. Y. Zhu P. Li S. Chen S. Y. Jiang X. H. Xu H. (2014). Rhamsan gum production by *Sphingomonas* sp. CGMCC 6833 using a two-stage agitation speed control strategy. Biotechnol. Appl. Biochem. 61, 453–458. 10.1002/bab.1185 24354661

[B61] Zhang Z. Chen H. (2010). Fermentation performance and structure characteristics of xanthan produced by *Xanthomonas campestris* with a glucose/xylose mixture. Appl. Biochem. Biotechnol. 160, 1653–1663. 10.1007/s12010-009-8668-y 19459070

[B62] Zhang J. Dong Y. C. Fan L. L. Jiao Z. H. Chen Q. H. (2015). Optimization of culture medium compositions for gellan gum production by a halobacterium *Sphingomonas paucimobilis*. Carbohydr. Polym. 115, 694–700. 10.1016/j.carbpol.2014.09.029 25439950

